# A Cylindrical Grip Type of Tactile Device Using Magneto-Responsive Materials Integrated with Surgical Robot Console: Design and Analysis

**DOI:** 10.3390/s22031085

**Published:** 2022-01-30

**Authors:** Yu-Jin Park, Eun-Sang Lee, Seung-Bok Choi

**Affiliations:** 1 Korea Initiative for Fostering University of Research and Innovation, Department of Mechanical Engineering, Inha University, Incheon 21999, Korea; eugene5059@inha.ac.kr; 2 Department of Mechanical Engineering, The State University of New York, Korea, Incheon 21985, Korea; 3 Department of Mechanical Engineering, Industrial University of Ho Chi Minh City, Ho Chi Minh City 70000, Vietnam

**Keywords:** magnetorheological (MR) materials, tactile device, repulsive force, stiffness of human tissue, surgical robot, cylindrical grip, magnetic analysis

## Abstract

This paper proposes a cylindrical grip type of tactile device that is effectively integrated to a surgical robot console so that a surgeon can easily touch and feel the same stiffness as the operating organs. This is possible since the yield stress (or stiffness) of magnetic-responsive materials can be tuned or controlled by the magnetic field intensity. The proposed tactile device consists of two main parts: a magnetorheological elastomer (MRE) layer and a magnetorheological fluid (MRF) core. The grip shape of the device to be positioned on the handle part of the master of the surgical robot is configured and its operating principle is discussed. Then, a couple of equations to calculate the stiffness from the gripping force and the field-dependent yield stress of MRF are derived and integrated using the finite element analysis (FEA) model. After simulating the stiffness of the proposed tactile device as a function of the magnetic field intensity (or current), the stiffnesses of various human organs, including the liver and heart, are calculated from known data of an elastic modulus. It is demonstrated from comparative data between calculated stiffness from human tissues and simulated stiffness from FEA that the proposed tactile device can generate sufficient stiffness with a low current level to recognize various human organs which are significantly required in the surgical robot system.

## 1. Introduction

### 1.1. Literature Review

Surgical robots were commercialized about 20 years ago, and robotic surgery has achieved remarkable development for nearly 10 years by accumulating data and technological developments. Robotic surgery and laparoscopic surgery are known as surgeries with less burden on patients since it enables operations with minimal invasiveness to be performed [1,2,3,4,5]. In conventional open surgery, a significant surgical site is required when the operation needs to be performed deep inside an organ. Human organs are complicatedly entangled, so it takes time to secure a field of view. Thus, laparoscopic surgery was introduced, and the method of inserting an endoscope can secure a field of view. It became possible to examine and operate on the abdominal cavity and the inside of the abdominal cavity. Furthermore, robotic surgery was introduced as a more precise and advanced surgical method. During robot surgery, imaging and visualization technology and contact force sensing and control technology have enabled tissue palpation in the manipulator [6]. Robot surgery minimizes damage to the affected area, and quick recovery after surgery minimizes patient downtime [7,8,9]. As this type of operation is performed by controlling a robot arm, it is difficult for the surgeon to interact directly with the patient. It is an operation that requires careful attention and a lot of skill. Numerous machines surround the patient on the operating table. The surgeon who operates is located in the console part of the robot. The operation is performed at a considerable distance from the patient. The surgeon operates and monitors the console unit simultaneously. When performing surgery, the surgeon relies on visual information to determine the operation of the robotic arm [10,11,12]. This is related to the proficiency of robotic surgery. Surgeons also argue that, unlike open surgery, robotic surgery requires more caution because it does not directly see the patient’s condition and it relies heavily on visual aspects because the affected area cannot be touched [13]. When the surgical site is narrowed and the camera view is inevitably narrowed, the amount of information delivered to the surgeon is even smaller [14,15]. This occupies a large part of the disadvantages of surgical robots. A surgery space in the abdomen is created with gas to secure a field of view, and then, surgical instruments are inserted. A camera is inserted together to communicate the condition inside the abdominal cavity to the surgeon. The surgery robot’s master controller is a pinch type, and very precise operation is possible. It is not easy to operate and requires a lot of skill. Recently, to aid the problems surgeons have raised, 3D images were developed and applied to the technology to deliver them to surgeons in three dimensions [14,15]. This is a case of improving the quality of information delivered by synthesizing images from various angles. However, it is still hard to enhance visual information, and it is not very relevant to surgeons who operate directly. Since the method of operation is not close to the patient, it can also cause poor judgment in emergencies.

Various simulations exist to increase surgeons’ operation accuracy and help them become familiar with it. Recently, various subtask devices have been developed that provide a virtual environment for surgeons to practice before performing robotic surgery [16]. The most widely used instrument, the Da Vinci Research Kit, originated at Johns Hopkins. It is possible to practice by composing an environment with materials similar to the tissues of humans. Even though such equipment has been developed, it is essential to learn the sense of the hand because it is necessary to perform microscopic surgery depending on the visual information displayed by the camera in the affected area. Additional sensory transmission is required to increase the success rate of surgery achieved from such a limited view. Even the most widely used da Vinci robot has not yet applied a haptic feedback system [17,18]. Therefore, if RMIS can deliver information on tactile sensation to the surgeon in real time during surgery, this problem will be solved to some extent. As a result, it is expected that applying the proposed haptic system can help surgeons to make immediate decisions and improve their surgical quality and accuracy when performing robotic surgery [19,20,21,22,23,24,25]. Haptic systems could be essential for surgeons who demand precision in their hands [23,24,25]. As the patient’s real-time information is delivered to the surgeon, it can help them make accurate and quick decisions. The internal state of the human body is very diverse. In the case of an undetected tumor, the tissue stiffness may differ from that of the surrounding area. Still, the problem may not be noticed unless you directly touch the deeply located area. These problems can be resolved to some extent with tactile feedback. It should be possible to change the tactile sense of various organs and tissues in real time with a single tactile feedback device against various tissues in the human body. Therefore, considering these points, various types of tactile transmission devices have been studied. As mentioned above, the most commercialized tactile feedback system is the vibration feedback system [26,27]. It is possible to transmit the tactile feeling by the intensity of the vibration. However, this system is a type that is often used as a warning message about external stimuli.

It is known that the vibration feedback system utilizing piezoelectric material is also a good option for use as a tactile device. As per several references, various human tissues and organs have the characteristics of viscoelastic materials. Therefore, in order to transfer more realistic information regarding the viscoelastic properties to surgeons, a tactile device which has similar or the same properties as human tissues and organs should be utilized to achieve high surgical quality and safety. However, it is very difficult to implement viscoelastic properties via the vibration feedback system due to the viscous effect that yields a time delay. Thus, it is not suitable for application to the tactile device associated with the console part of the surgical robot. Other systems include piezoelectric technology [28]. This is a method that delivers tactile sensation according to various arrangement methods. A wide range of forces and sufficient forces can be applied [28]. However, it is not suitable to express the state of tissue inside the human body to transmit simple force. Since the human body has viscoelastic properties, a method that can express these properties together is more suitable. Therefore, a pneumatic tactile transmission device has been proposed to deliver viscoelastic properties together [29]. However, in the case of pneumatic pressure, there is a lack of expression of the state of tissues with incompressible characteristics because compressible gas is entered. To supplement these points, many tactile devices using magnetorheological materials have been recently proposed in RMIS (Robot-assisted Minimally Invasive Surgery) [30,31,32,33,34,35,36,37]. A lot of research has been carried out on the development of Haptic Master using MR materials [30,38]. Additionally, the MR Tactile Cell Device, a method that delivers haptic information directly to the surgeon’s hand, has been proposed [39,40,41,42,43]. Since the magnetorheological-based tactile device changes the yield stress by adjusting the magnetic field strength, it is possible to represent the organ characteristics of various human tissues with a single sample.

### 1.2. Proposed Design Methodology

Most of the previous papers focused on designing and manufacturing a single object, and no specific application plan was presented [42,43]. In the case of the MR material-based device, since it is made of one tactile cell, another part of the touch sensor can be made without directly grafting it to the robotic surgical environment. It is very inconvenient to simultaneously detect the stiffness generated by the human body from the touch sensor. Therefore, to improve this, there is a need for a tactile device that can be applied to a surgical environment. The main technical contribution of this paper is to suggest a new type of tactile device which can be easily applied to the controller of an existing surgical robot console. The currently commercialized haptic system needs to be modified for application to the console unit of a surgical robot. Several haptic systems’ in-game controllers are widely used, as these are the first cases to be commercialized and are popular [44]. A haptic response is generated and delivered to the human hand by controlling the strength and speed of vibrations. These haptic devices are optimized and applied to the user’s grip. As such, the haptic part is designed to consider the area touched by a person’s palm and the grip of the finger. This form is also applicable to the controller of the console unit of a surgical robot. Therefore, it is possible to design a new type of tactile device according to the grip feeling of the hand. The goal of the proposed tactile device is to realize the same stiffness from the tactile device after sensing the force in the end effector of the abdominal cavity for the stiffness of various tissues generated in laparoscopic surgery or robotic surgery. To achieve this goal, a cylindrical grip type of tactile device consisting of a magnetorheological elastomer (MRE) layer and a magnetorheological fluid (MRF) core is devised, and its stiffness variance by the magnetic field intensity (or current) is simulated from the finite element method (FEM). To validate the effectiveness and feasibility of the proposed tactile device, the stiffnesses of various human organs are calculated based on the modulus values. It is shown from the data comparison between a simulation and calculations that the proposed cylindrical tactile device gripped by the master handle can generate sufficient stiffness with a low current to cover most of the human tissues. It is noted here that the proposed tactile device is expressed by MRTGC (Magnetic Response Tactile Grip Control) from now on.

## 2. Design Concept of MRTGC

**Figure 1:**
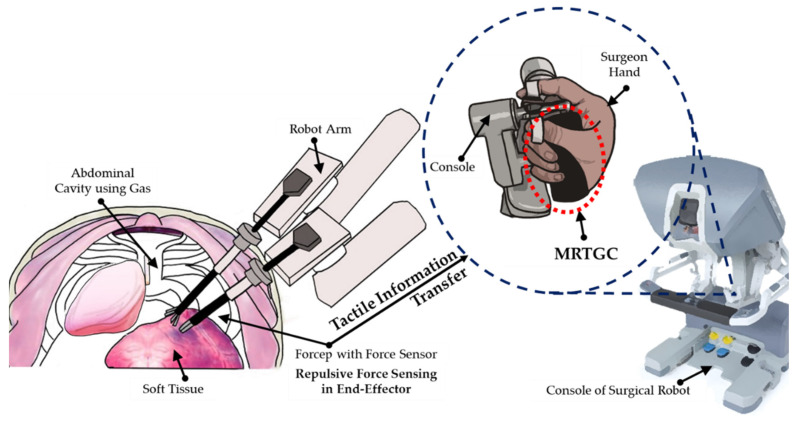
Schematic diagram of the overall control system integrated with the proposed MRTGC on the console of a surgical robot.

Figure 1 shows a schematic diagram of the overall console control system associated with the proposed MRTGC. The end-effector senses the stiffness to transmit tactile information during robot surgery. Since the stiffness must be sensed and transmitted in real time, the end effector must be configured to enable force sensing. Based on the sensed information, the current is applied to MRTGC to adjust the stiffness so that the surgeon can realize the same stiffness from the tactile sensor as that of the operated human organ (or tissue). As shown in the figure, the cylindrical shape of the tactile device can be easily grasped by fingers for the pinch grip, making it comfortable to hold. Thus, the main goal of this study is to make the controllable area wider using MRF so that the surgeon can feel the stiffness by holding the device. In order to achieve this goal, the magnetic field analysis of the device is carried out by considering the relationship between the yield stress of MRF (τ) and the magnetic field intensity (H). On the other hand, it is carried out to calculate how much the normal force can be changed due to the change in the stiffness of MRF. In this work, a three-dimensional model is created, and the stiffness is obtained from the finite element analysis (FEA). The human control group is then calculated by assuming that the obtained value is the same area as the tactile device. The stiffness of various human organs is calculated from the known data of the elastic modulus. Consequently, to demonstrate the feasibility of the proposed MRTGC, the simulated values of the stiffness from FEA and the calculated stiffness from the elastic modulus of human tissues are compared and analyzed.

**Figure 2:**
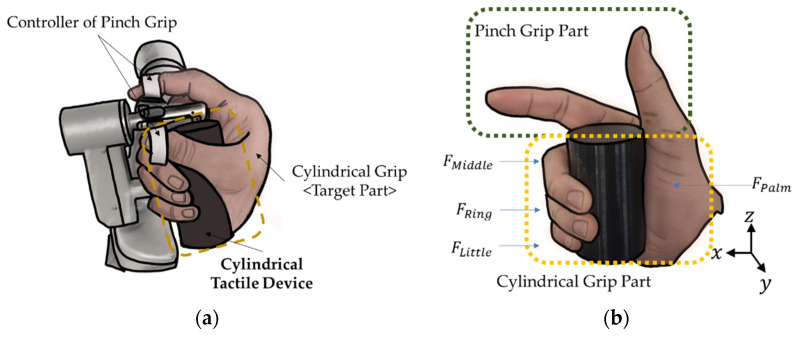
Cylindrical type tactile device combined with the pinch grip; (**a**) MRTGC integrated with the existing console, (**b**) free body diagram of the gripping motion.

It is known that there are two types of grips for surgical robots: the pinch grip type and the power grip type. The power grip type [45] is also called a cylinder grip. The advantages and disadvantages of each are as follows. In the case of the pinch grip (precision grip) [45], the accuracy is high, but it is not comfortable to use. If the operator is inexperienced, it can be a rather dangerous situation. Therefore, it is a type that requires a lot of skill. Additionally, since it is used by hanging a finger, the area to which the tactile device can be applied is small. On the other hand, the power type is easy to control with sufficient comfort. However, it has relatively low accuracy. When modifying the haptic application part of the joystick, it is useful to use the cylindrical type since the generated stiffness from the device can be easily delivered to the hand (or fingers) wrapped around the device. However, the pinch grip can provide higher precise manipulation. Therefore, in this work, a combination of the two types is adopted. Figure 2 presents the MRTGC integrated with the existing pinch type console. Specifically, Figure 2a shows the conceptual design in which the fingers and palms, except for the thumb and index finger, which are pinch grips, comfortably grip the MRTGC. The part corresponding to the proposed MRTGC can exclude the index finger and thumb of the $F_{Palm}$ and pinch controller parts. The remaining fingers ($F_{Middle}$, $F_{Ring}$, and $F_{Little}$) can be newly composed as a free body diagram, shown in Figure 2b. The dimension (or size) of the MRTGC is initially designed in consideration of the area of the fingers and palm remaining under the cylindrical grip.

From the free-body diagram shown in Figure 2b, the force balance equation is obtained when the hand grips the MRTGC. The stiffness generated by the device when holding the device by hand is expressed as follows ($F_{Cylindrical\;Grip}$):

(1) $$F_{Middle}+F_{Ring}+F_{Little}=F_{Palm}\;\left|F_{Middle}+F_{Ring}+F_{Little}+F_{Palm}\right|=F_{Cylindrical\;Grip}$$

In this equation, $F_{Cylindrical\;Grip}$ means the force holding the cylinder by the hand. Therefore, based on the *x*-axis, the absolute value of the force that the fingers and palm are pressing on both sides is taken, and the added value is displayed when the cylinder is held. The figure below shows the free body diagram of the force applied to the cylinder in the *x*-axis direction. When the hand applies the force to this cylinder to hold it, the force received by each part will be applied as shown in the figure below, and the formula can be expressed as follows: $F_{Middle}+F_{Ring}+F_{Little}$ = $F_{Palm}$. Therefore, Formula (1) is obtained by assuming that $F_{Cylindrical\;Grip}$ is the total force applied to the cylinder. It is noted here that the sum of all forces is zero with respect to the center line of the cylinder, and hence, the abosolute value can be calculated. On the other hand, a function of force concerning the magnetic field $F_{MRTGC}\left(H\right)$ is defined which is equal to $F_{Cylindrical\;Grip}$. Against this gripping force, MRTGC must transfer the tactile information received from the end-effector by changing the stiffness according to the magnetic field in the part where the hand wraps around it. Therefore, the stiffness of MRTGC (force according to human grip) generated from the field-dependent yield stress of MRF can be expressed by the following equation:

(2) $$F_{Cylindrical\;Grip}=F_{MRTGC}\left(H\right)$$

It is remarked here that the stiffness of MRTGC due to gripping or the yield stress can be obtained through structural analysis using the finite element method (FEM) associated with a 3-D model. This is carried out in Section 4.

**Figure 3:**
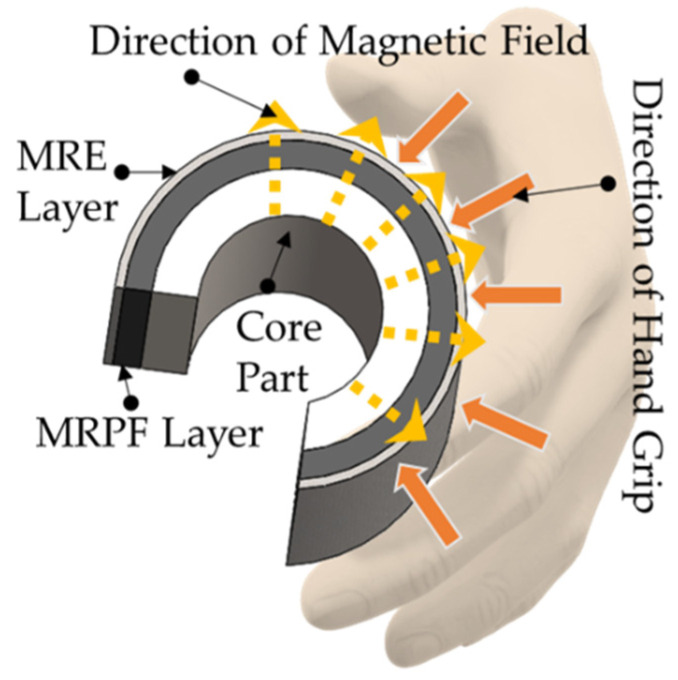
The structural configuration of the proposed MRTGC.

Figure 3 shows the specific components of MRTGC with the directions of the handgrip and magnetic field. It is known that the behavior of MRF can be broadly divided into three types: flow, shear, and squeeze mode. In this study, the squeeze mode motion is dominant from the directions of the gripping and field generation. The stiffness of MRTGC mainly comes from the field-dependent yield stress of MRF soaked into the polyurethane foam (in short, MRPE), while the MRE layer is used as a cover since it is flexible and easy to seal for the leakage protection of MRF. The stiffness of the MRPF layer is also changed according to the formation of the magnetic field, and as a result, various tactile sensations can be delivered accordingly to the gripping hand (or fingers). Consequently, the primary materials to compose the shape of MRTGC shown in Figure 3 are as follows: MRF, MRE, and polyurethane foam.

## 3. Characteristics of Magnetic-Responsive Materials

### 3.1. Magnetorheological Fluid

**Figure 4:**
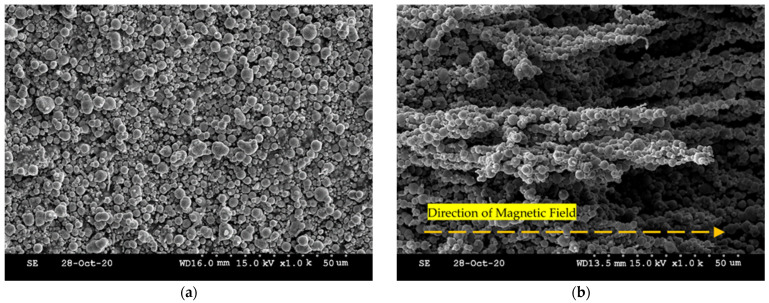
The micro-behavior of particle motion in MRF: (**a**) off a magnetic field, (**b**) on a magnetic field.

In general, a magnetorheological (MR) material refers to a material whose properties change depending on the presence or absence of a magnetic field. Among several MR materials, MRF is the most popular for research and commercialization since it has several salient benefits: a fast response time, reversible behavior, high yield stress, and excellent control performance. MRF is a magnetorheological substance containing fine iron particles in a base silicone oil [46]. The properties of MRF depend on the type of oil and the weight of the iron particles. For example, the higher the particle concentration, the higher the yield stress of MRF. Figure 4 presents the behavior of MRF with and without the magnetic field (MRF-132DG, Lord Corporation, Cary, North Carolina, USA). Figure 4a shows that the particles in the base oil are randomly distributed when it is turned off. When the magnetic field is applied, the particles form a chain-like structure along the magnetic field lines and appear to have a certain direction, as shown in Figure 4b. Depending on the magnetic field, this chain is formed and released, changing the characteristics of MRF. As the strength of the magnetic field increases, the chain becomes denser and more robust [47].

**Figure 5:**
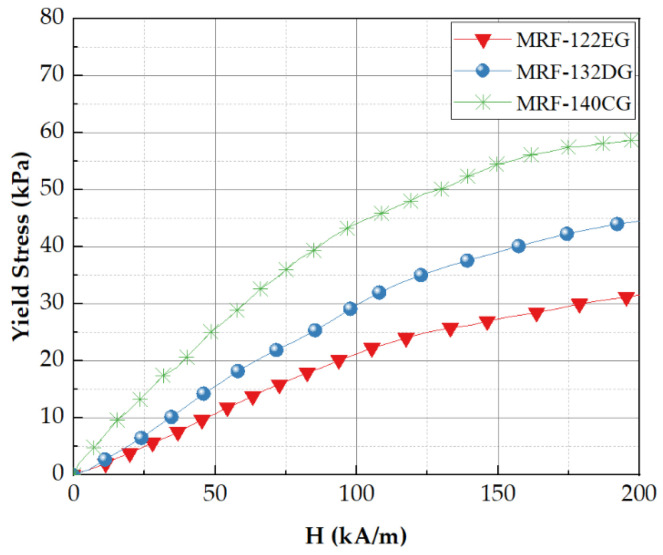
The field-dependent yield stress of various MRFs.

The MRF used for the calculation of the stiffness of MRTGC is MRF-132DG, which is suitable for mimicking human tissues with low yield stress. It is seen from Figure 5 that MRF-132DG is positioned between two other MRF models, indicating an appropriate yield stress with a relatively low magnetic field. It is noted here that MRF models used for Figure 5 are ones commercialized by Lord Company, USA. In the previous studies on tactile devices using MRF, the very large magnetic core of the electromagnet applied to the tactile cell was a critical problem due to the requirement of a high magnetic field [42,43]. Since the newly designed core in this work must fit the cylindrical grip of a person’s hand, the core must be elongated and smaller. It is remarked that MRF-122EG is a good candidate in terms of power consumption, but the stiffness range from the yield stress is limited. In terms of stiffness efficiency, MRF-140CG seems to be the best, but it requires too high a magnetic density. The relational polynomial of τ(H) can be obtained using the data shown in Figure 5. Here, H indicates the magnetic intensity. The linear least-squares method is applied to find the coefficients of H using a curve fitting software of Matlab. The polynomial is later used to find the stiffness of the designed tactile device in the magnetic field analysis. The polynomial equation achieved from the curve fitting is given by the following equation:

(3) $$\tau \left(H\right)=-6.773H^{5}\times 10^{-11}+5.961H^{4}\times 10^{-8}-1.805H^{3}\times 10^{-5}+0.001463H^{2}+0.287H-0.7213$$

### 3.2. Magnetorheological Elastomer and Polyurethane Foam

**Figure 6:**
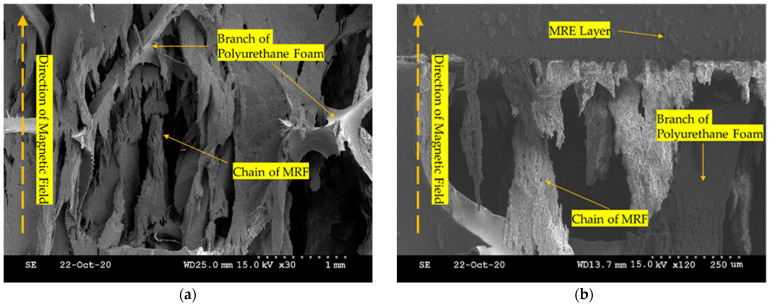
The micro-structure behavior of the tactile device component interacting adjacent structures; (**a**) MRF chain + branch of polyurethane foam, (**b**) MRE layer + MRF chain + branch of polyurethane foam.

MRE is also one of the magnetorheological materials whose stiffness changes due to the interaction of CIP (Carbonyl Iron Particle) particles distributed between the rubber-like matrix according to the strength of the magnetic field. The strength of the magnetic field controls the stiffness of the MRE. However, to feel the change in the stiffness of MRE, effective results can only be seen when it is placed under high-frequency vibration. In this tactile design, MRE is used to help the formation of the chain-like structures by wrapping both MRF and polyurethane foam. Thus, the contribution of MRE to the stiffness change of MRTGC is small and hence neglected. Figure 6 presents the micro-structure interacting among the MRE layer, MRF, and polyurethane foam under the magnetic field. The polyurethane foam is adopted as a structure that can help form the chain of MRF. This foam can be manufactured with various stiffness, and a suitable material is selected in this work considering the stiffness effect of MRTGC. MRF is well absorbed, and even when the magnetic field is applied, it can perform the role of a frame well enough. It is seen in Figure 6a that the CIP particles of MRF are formed from the branch of polyurethane foam. Figure 6b shows the interacting motion among the materials used for MRTGC. It is remarked here that using MRE as a cover layer is more effective in forming the magnetic field than using the traditional silicon rubber. As the strength of the magnetic field increases, the MRF chain between pores becomes stronger by connecting the branches of foam. As a result, the MRF chain connecting the branches of polyurethane foam causes the stiffness change in the tactile device.

## 4. Yield Stress Analysis of MRTGC

**Figure 7:**
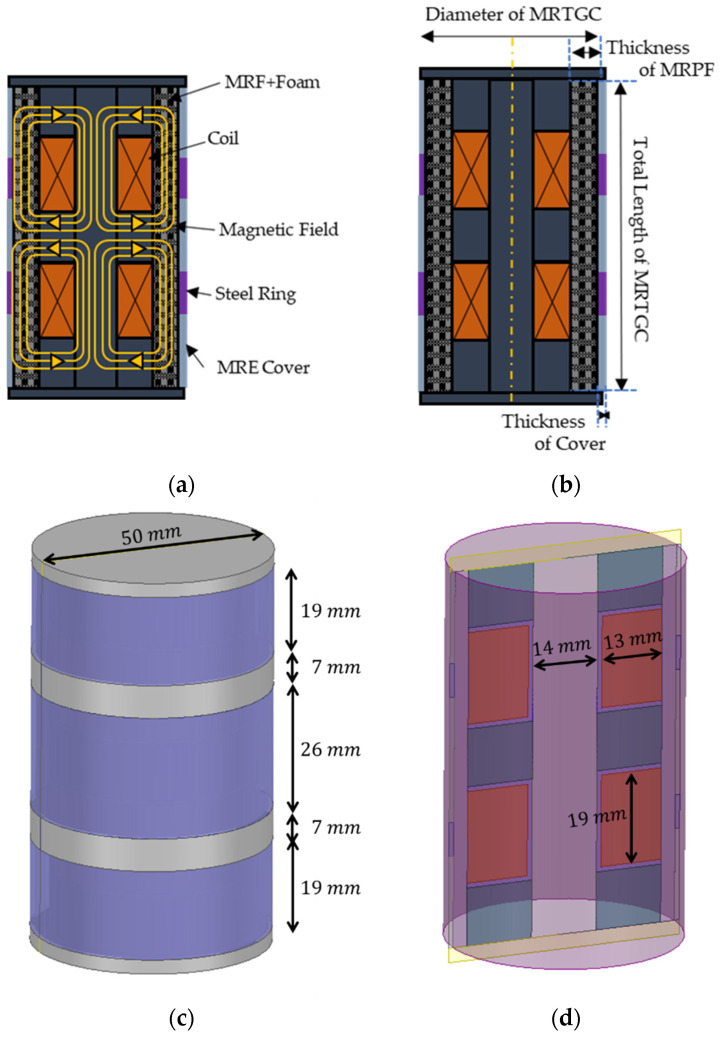
Magnetic core model of the tactile device: (**a**) cross-section and magnetic field formation scheme, (**b**) main design parameters, (**c**) 3D structure model of tactile device, (**d**) cross-section of 3D structure model.

Figure 7a presents the core location and magnetic field area of the cylindrical type of tactile device. Two coils are located to form the magnetic core, and the direction in which the current is applied has a strong magnetic field, resulting in the effective pole. MRF-132DG absorbed in polyurethane foam is located on the outside of the coil. MRE and steel ring parts are wrapped around this and fix it. The main design parameters of MRTGC are denoted in Figure 7b. The 3D model of the overall shape is shown in Figure 7c. A cross-section of the 3D model is shown in Figure 7d. Considering the size of the handgrip, the total length is chosen to be 70 mm, and the diameter is 55 mm. In addition, the thickness of the inner MRFP is 3 mm. The thickness of the cover (MRE and Steel Ring) is 1mm. The number of coil turns of the magnetic core is 600. The yield stress of MRTGC is given by the following equation:

(4) $$\tau_{yield}{\left(H\right)}_{effective}=\int_{0}^{v}\left(\overset{n}{\underset{0}{\sum }}\tau_{yield}\left(H\right)\right)dv$$

It is clear that the $\tau_{yield}$ is a function of the magnetic field intensity *H*. Therefore, according to the current input, the effective area can be calculated. More specifically, the effective area value for each magnetic field strength is calculated by integrating the value obtained by adding the *H* value of each node. v is volume. Then, the $\tau_{yield}$ can be calculated according to the current input using Equation (4). The relevant process is also performed together in the magnetic field analysis to calculate the *H* value.

**Figure 8:**
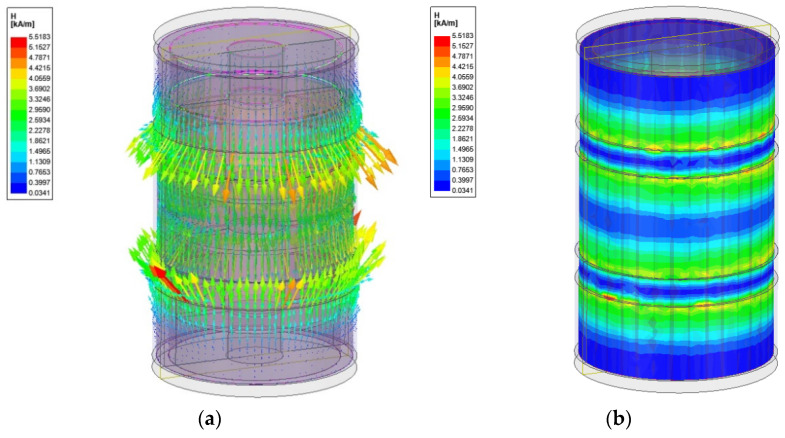
Magnetic field analysis result of the designed tactile device; (**a**) vector expressions of magnetic intensity at the effective surface in the cross-section, (**b**) magnitude contour expressions of magnetic intensity at the effective surface in the cross-section.

**Figure 9:**
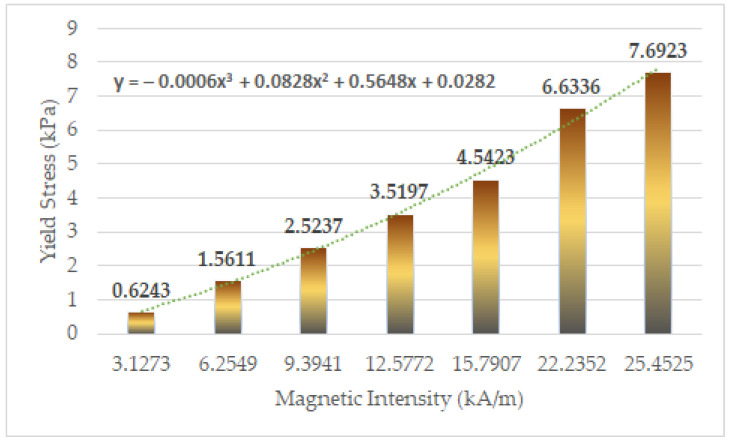
The relationship between the yield stress and magnetic field achieved from the magnetic field analysis (τ(y)−H).

Figure 8 presents the magnetic field analysis result of the designed tactile device. Figure 8a shows the vector express of the magnetic intensity values, while Figure 8b shows the magnitude express of the magnetic intensity values. The H (magnetic intensity) value is used to obtain the stiffness formed according to the current input in the tactile device. Figure 8 shows the simulation results for an input value of 0.35 A. The H value is calculated through magnetic field analysis after simulating 0.05 A (current) intervals when 0.4 A is applied. The Ansys Maxwell program (Ansys Incorporation, Canonsburg, PA, USA) is used for the simulation. Each core part is wound 300 times, and an external circuit is formed to gradually supply current within a certain time. The maximum current value is 0.35 A. The mesh count is created with the maximum set to 30,000. Figure 9 presents the bar graph showing the yield stress versus the magnetic field strength. Influenced by the curve-fitting polynomial of MRF-132DG, the yield stress is expressed by the non-linear polynomial. Table 1 shows the values calculated using Equations (3) and (4). Using Equation (3), the magnetic field strength in the effective area is firstly calculated according to the current. The yield stress according to H intensity is then calculated using Equation (4). These values are used to calculate the stiffness through FEA in the subsequent section.

## 5. Stiffness Analysis

**Figure 10:**
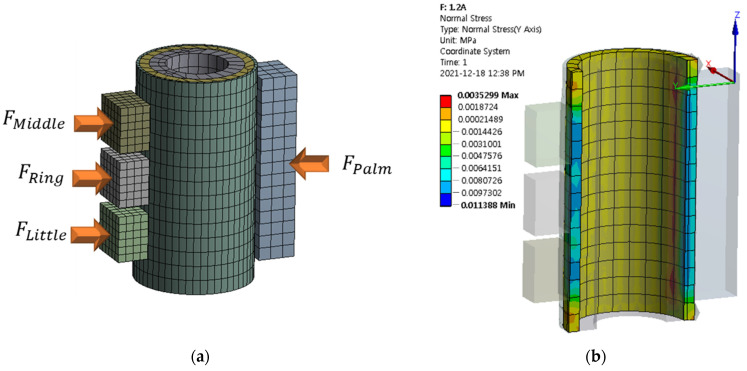
The finite element model of MRTGC with the handgrip: (**a**) 3D model of meshed MRTHC, (**b**) cross-section of normal force generated when hand deformation occurs in MRTGC section.

In order to determine the field-dependent stiffness, the structural analysis of MRTGC is undertaken, utilizing the finite element method (FEM). The Ansys Workbench program (Ansys Incorporation, Canonsburg, Pennsylvania, USA) is used for the simulation. To input each human source into the engineering data, all tissues are assumed to be incompressible. Therefore, when entering the elastic modulus, all Poisson ratios are fixed at 0.49. Since the 3D model is not a complex structure, the mesh is created as an automesh as a coarse type. The number of created nodes is 15,479, and the number of elements is 2385. The model used in this work is shown in Figure 10, and $F_{Middle}$, $F_{Ring}$, $F_{Little}$, and $F_{Palm}$ in Equation (1) are applied as shown in Figure 10a. The stiffness is calculated assuming that these forces press the cylinder by 1 mm from both sides of the cylinder. For $F_{Cylindrical\;Grip}$, all normal forces in which MRPF occurs are calculated based on the direction in which the force is applied (based on the *y*-axis in the analysis). The normal force generated in the MRPF when the grip is applied is shown in Figure 10b. The values calculated through FEM simulation are presented in Table 2. It is remarked here that in this simulation, the maximum current is limited by 0.35 A, considering safety issues with regard to the gripping status.

**Figure 11:**
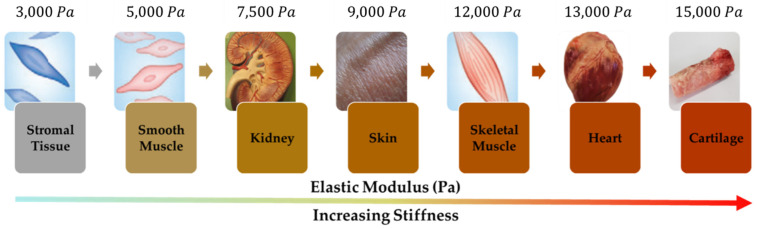
Elastic modulus of various types of human tissues [48,49].

**Figure 12:**
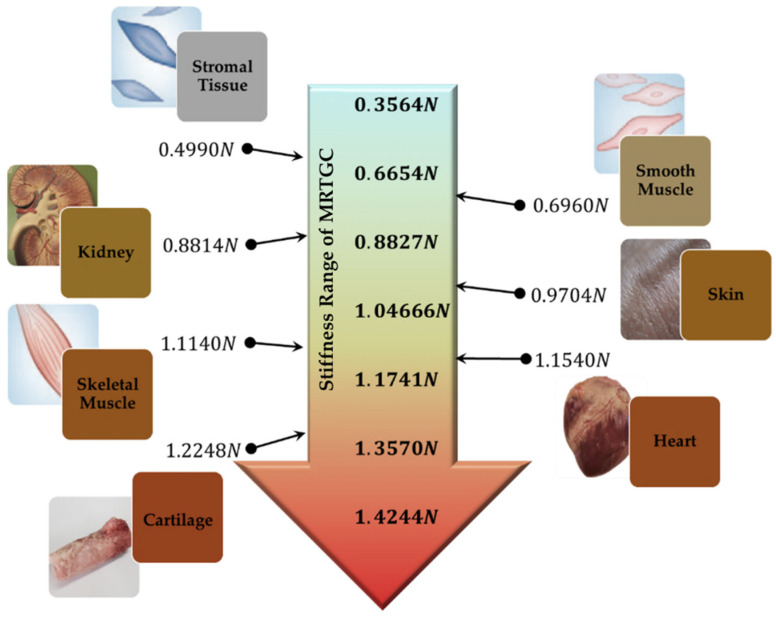
Comparison between MRTGC of stiffness calculated by simulation results and human tissue data.

Now, material data of various human organs are required to calculate the stiffness and compare with the simulated data from FEA of the designed MRTGC. Therefore, the structural analysis is carried out using the same method as before using the elastic modulus of major human organs. The elastic modulus is calculated using the cylindrical grip model shown in Figure 11. The elastic modulus of various human organs and tissues are given in the order of size, and seven types are used for the calculation, from stromal tissue with low modulus to cartilage with high modulus. In addition, in the calculation of the stiffness of human organs, the Poisson’s ratio of 0.49 is used for tall tissues, assuming that those are incompressible. Table 3 gives the human’s stiffness values obtained from this process. Figure 12 compares the field-dependent stiffness generated from MRTGC with the human organs’ ones calculated based on the elastic modulus of each organ. It is clearly seen that the control group of human organs’ stiffness are included within the stiffness spectrum, which are generated from the proposed MRTGC. This shows that the stiffness of MRTGC itself is adjusted according to the input value of the designed current, and it is possible to reproduce the sensed stiffness inside the human body and transfer it to the surgeon. Specifically, with the magnetic field strength of 0.05 A to 0.35 A applied to MRTGC, the stiffness is altered from 0.3564 N to 1.4244 N, respectively. Both the stromal tissue with the lowest stiffness and cartilage with the highest stiffness is mimicked by the proposed MRTGC, which is integrated with the controllable console of surgical robot systems.

## 6. Conclusions

In this paper, a new tactile device that can be applied to the controller of a surgical robot was proposed, and its field-dependent stiffness was simulated using the finite element analysis (FEA). A cylindrical type of tactile device utilizing magnetorheological materials MRTGC was devised and designed so that a surgeon can easily grip the device during an operation. The main components of this device consist of MRF absorbed into polyurethane foam with magnetic cores and MRE as cover to provide smooth magnetic circuit formation and prevent the leakage of MRF. The magnetic intensity of the conceptually designed device was calculated based on the effective method using magnetic analysis via the Ansys Maxwell program (Ansys Incorporation, USA). In this analysis, a simplified 3D model was used considering external gripping force. Then, the field-dependent stiffness of MRTGC was simulated using the material data of MRF: the relationship between the yield stress and the magnetic field intensity. As for the comparative group, the repulsive forces of various human organs were also calculated with the data of the elastic modulus using the same FE model and process. Then, a comparison of the stiffness between the simulated values from the proposed MRTGC and calculated values of human organs was made. It was shown that the stiffness of MRTGC can include most human organs. More specifically, MRTGC can generate stiffness from 0.3564 N to 1.4244 N by applying a current from 0.05 A to 0.35 A. This range includes both stromal tissues with the lowest stiffness (0.4990 N) and cartilage with the highest stiffness (1.2248 N).

It is finally remarked that in order to actually implement the proposed tactile device in surgical robotic systems, some issues need to be explored in the future. These issues include an optimal design to adapt it to an existing console of a surgical robot by focusing on the geometry minimization and joint connection between the console and the surgeon’s gripping hand, an experimental validation of the data accuracy and repeatability as a function of time by focusing on the effect of the magnetic field intensity and surgical motion movement, an establishment of a feedback loop for the stiffness communication between the proposed device and the organs to be operated on by focusing on the surgeon’s training process, an integration with the existing joystick haptic system of a surgical robot by focusing on the surgeon’s feeling from the operating organs and the stiffness felt from the proposed device, and an extension of the proposed device which can generate both the stiffness (force) and the torque, which frequently occurs during robot surgery by focusing on the configuration modification of the cylindrical type.

## Figures and Tables

**Table 1:** The calculated values of *H* and yield stress generated from the effective area of MRTGC.

Magnetic Field Strength (Input Value of Current) Unit: A	Magnetic Intensity H (kH/m)	Yield Stress τ(H) kpa
0.05	3.1273	0.6243
0.1	6.2549	1.5611
0.15	9.3941	2.5237
0.2	12.5772	3.5197
0.25	15.7907	4.5423
0.3	22.2352	6.6336
0.35	25.4525	7.6923

**Table 2:** The simulated value of stiffness of MRTGC.

Magnetic Field Strength (Input Value of Current) Unit: A	Stiffness (N)
0.05	0.3564
0.1	0.6654
0.15	0.8827
0.2	1.0466
0.25	1.1741
0.3	1.3570
0.35	1.4244

**Table 3:** Calculated stiffness of human tissues from the elastic modulus.

Human Tissue	$F_{tissue}\;\left(N\right)$
Stromal Tissue	0.4990
Smooth Muscle	0.6960
Kidney	0.8814
Skin	0.9704
Skeletal Muscle	1.1140
Heart	1.1540
Cartilage	1.2248

## Data Availability

Not applicable.
